# Sleep Timing Across the Lifespan of Australian Adults

**DOI:** 10.3390/clockssleep7010016

**Published:** 2025-03-20

**Authors:** Gabrielle Rigney, Matthew Browne, Charli Sargent, Michele Lastella

**Affiliations:** 1 Appleton Institute, School of Health, Medical and Applied Sciences, Central Queensland University, Adelaide, SA 5034, Australia; c.sargent@cqu.edu.au (C.S.); m.lastella@cqu.edu.au (M.L.); 2 School of Health, Medical and Applied Sciences, Central Queensland University, Bundaberg, QLD 4670, Australia; m.browne@cqu.edu.au

**Keywords:** bedtime, wake-up time, adults, sleep behaviour, sleep duration, health, mood

## Abstract

The aim of this study was to examine sleep timing across the lifespan of Australian adults. A cross-sectional design was used to collect information on subjective sleep timing from 1225 participants (52.3% female) during a telephone interview. The participants were aged from 18 to over 80 and were grouped according to their age using 10-year increments (e.g., 18–29 y, 30–39 y, etc.). There was a diverse distribution across the lifespans, with the largest proportion of participants being from the 60–69 age group (22.8%). Participants were predominantly from New South Wales, Queensland, and Victoria. Younger adults reported going to bed later (*p* < 0.001) and waking up later than other age groups (*p* < 0.001). Wake times were earliest during middle adulthood (*p* < 0.001). There was no significant age effect on the minimum sleep required for good health (*p* = 0.159) and only a marginal decrease with age in the amount of sleep required to maintain a good mood (*p* = 0.041). In conclusion, these findings highlight significant variations in sleep timing across younger, middle-aged, and older Australian adults. The current findings could inform future Australian sleep health campaigns, in which the goal is to provide targeted strategies for age groups across their lifespans.

## 1. Introduction

Sleep is essential to maintain optimal health and well-being throughout our lives. There are predictable developmental changes that occur in sleep behaviour throughout the lifespan [1]. Many major developmental changes occur throughout childhood and adolescence, with sleep requirements becoming more stable in adulthood [1]. However, various psychosocial and other lifestyle factors that we encounter throughout different stages of life (e.g., work, friendships and relationships, parenthood, primary caregiver roles, and retirement) are also strong determinants of our sleep behaviour [2].

Data from the Australian Sleep Health Foundation’s national survey examining the sleep health of Australian adults report that the average sleep duration on a weekday is approximately 7 h per night [3]. Current sleep recommendations state that adults aged 18–64 years should aim to obtain between 7 and 9 h of sleep per night, and adults over the age of 65 years are recommended to obtain between 7 and 8 h of sleep per night [4]. Australian adults are therefore just meeting the recommended sleep duration [2]. Differences in sleep duration across the lifespan were also identified in Australians; younger adults reported sleeping approximately 1 h longer on weekends than older adults [3]. However, sleep timing variables such as bedtimes and wake times were not reported. In order to improve sleep behaviour, understanding sleep timing is critical, as both bedtimes and wake times are modifiable behaviours.

In a recent meta-analysis examining age and sleep characteristics across the lifespan, sleep timing was suggested as an important way to characterise the effects of age on sleep [5]. Wallace et al. [6] examined age trends in sleep across the lifespan, using both actigraphy and self-reported data from the Pittsburgh Lifespan Sleep Databank. The large sample (*n* = 1065) included participants aged between 10 and 87; however, they had fewer participants in the middle years of life (~40–~60 years). The key findings support what is known about sleep changes in adolescence, where bedtime and wake time are later, and during the twenties through to mid-thirties, both bedtime and wake time shift to an earlier time [6]. Meta-analyses and other studies report similar findings related to sleep timing across the lifespan [7,8,9]. Another large study exploring sleep characteristics across the lifespan of over 1.1 million people from the Netherlands, United States, and United Kingdom [7] identified differences in sleep behaviour between countries, highlighting the importance of understanding country-specific sleep behaviours. While there is evidence describing sleep timing across the lifespan more broadly, less is known about sleep timing across the lifespan specific to Australian adults.

Conclusions from the Australian Sleep Health Foundation survey acknowledged that sleep problems were prevalent in Australian adults and recommended that a greater focus on healthy sleep at a policy level was needed to increase public awareness about the importance of obtaining adequate sleep [3]. The impact of sleep on overall health and well-being is an important component to consider when raising awareness about improving sleep behaviour. The bidirectional relationship between sleep and health is well established. The consensus statement released by the American Academy of Sleep Medicine and the Sleep Research Society stated that a range of deleterious effects are associated with obtaining less than 7 h of sleep per night on a regular basis [10]. These include both physical health (e.g., weight gain, hypertension, stroke) and mental health (e.g., mood/depression) outcomes [10]. Gaining further insight into Australian adults’ perceptions of the sleep–health relationship would be useful when establishing sleep health campaigns.

The primary aim of the current study was to examine self-reported sleep timing behaviours in Australian adults across the lifespan. A secondary aim of this study was to explore how much sleep Australian adults perceive to be necessary to feel healthy and be in a good mood.

## 2. Results

**Figure 1:**
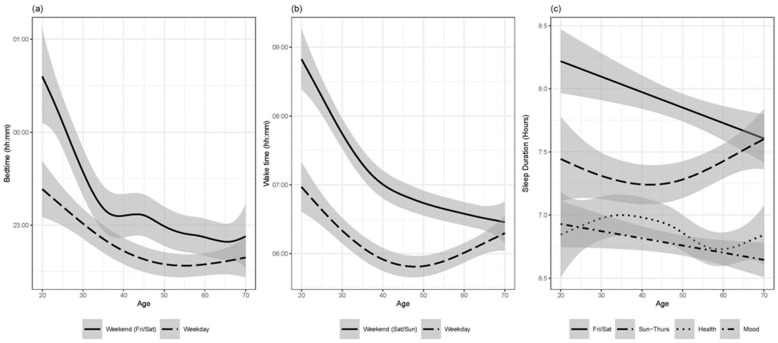
Panels (**a**–**c**) summarise the GAM fits of the various outcomes as a smooth function of age, with the 95% confidence intervals of the fit given by the shaded areas. (**a**) shows the effect of age on bedtime over the lifespan, and (**b**) shows the effect of age on wake time over the lifespan. (**c**) shows the effect of age on total sleep duration, as well as the sleep duration desired to maintain one’s health and mood.

Figure 1a–c summarise the GAM fits of the various outcomes as a smooth function of age, with 95% confidence intervals of the fit given by the shaded areas. Figure 1a shows the effect of age on bedtime over the lifespan, and Figure 1b shows the effect of age on wake time over the lifespan. Bedtime was significantly later on the weekends for younger people F(4.154) = 18.530, *p* < 0.001, with a similar but attenuated trend also evident for weeknights F(3.502) = 10.080, *p* < 0.001 (Figure 1). The wake time on weekends was latest for young people, and became markedly earlier with increasing age F(4.429) = 32.34, *p* < 0.001. Wake times on weekdays were slightly earlier in middle-aged adults compared to younger adults, and then became slightly later again in older age F(4.654) = 9.524, *p* < 0.001 (Figure 1).

Figure 1c shows the effect of age on total sleep duration, as well as the sleep duration desired to maintain health and mood. Sleep duration on weeknights (Sun–Thurs) decreased slightly through to middle age, and then increased afterwards up to 7.6 h in old age, F(2.83) = 5.08, *p* < 0.001 (Figure 1). However, sleep duration on the weekend tended to decrease linearly over the lifespan, F(2.78) = 3.27, *p* = 0.0186. There was no significant effect of age on the minimum amount of sleep people required to maintain good health, F(2.586) = 1.614, *p* = 0.159, and only a marginal decrease with age in the amount of sleep required to maintain a good mood, F(1.646) = 3.155, *p* = 0.0412.

## 3. Discussion

Changes in sleep timing were evident across the lifespan of Australian adults, similar to what has been reported in other countries to date [5,9]. Younger adults reported going to bed later and waking up later than other age groups, which was more pronounced on weekends than weekdays. These findings are consistent with a previous national survey exploring the sleep health of Australian adults, in which younger adults slept for 1 h longer on weekend days than older adults [3]. Wake times were earliest during middle adulthood, which may be reflective of lifestyle factors specific to this age range, such as being career-focussed and/or maintaining childcare responsibilities. By the age of 70, the difference in wake times for weekdays and weekends had dissipated, which likely reflects retirement age, where sleep behaviour is no longer influenced by work schedules [11].

Participants perceived that the minimum amount of sleep required to feel healthy and to be in a good mood was, on average, less than the amount of sleep they reported obtaining. Interestingly, the average minimum amount of sleep perceived to be required for feeling both healthy and being in a good mood was predominantly under 7 h across the lifespan, which is less than the current sleep recommendation target [4]. The decrease in sleep observed with age in terms of the minimum amount of sleep required to maintain a good mood indicates that younger adults perceived needing more sleep to maintain a good mood compared to older adults. This is in line with current research evidence, which indicates that the mood of older adults is less impaired than in younger adults following sleep deprivation [12]. Additionally, evidence suggests that older adults are more effective at regulating emotions in order to optimise their well-being than younger adults [13]. Given that significant age group differences for the minimum amount of sleep required to maintain a good mood were found, but significant changes across the lifespan were not observed when asked about the minimum amount of sleep required to feel healthy, this perhaps demonstrates that adults are more sensitive and aware of the role sleep has on their mood each day compared to their overall health.

The findings of the current study must be interpreted within the boundary conditions of its cross-sectional design. A cross-sectional design allows for comparison between cohorts of different ages but cannot reveal causal changes across the lifespan. The use of GAMs was beneficial due to their flexibility in modelling complex, non-linear relationships and their balance between accuracy and interpretability. A limitation of this study is the absence of an a priori power calculation. While we are confident that the large sample size was adequate to detect significant effects, future studies would benefit from conducting an a priori analysis to further validate sample size adequacy and enhance reliability. A further limitation is that the participants were asked to self-report their sleep timing behaviours, which could be inaccurate due to biases such as general recall and social desirability. A measure of sleep quality was not included in the current study, but is recommended in future studies to provide a comprehensive sleep assessment and improve research validity. Additionally, equal numbers of participants across all age groups were not obtained, with approximately 40% of the sample being in the 50–70 years age range. Interestingly, approximately 34% of Australia’s population is aged 50 and over [14], which reflects the ageing population. However, this imbalance between the age groups in our study limits the generalizability of the study’s findings. In the future, having equal numbers across all age ranges is recommended and objective measures of both sleep quality and quantity should be included.

The previous literature highlighted that country-specific differences in sleep characteristics across the lifespan exist [7], and therefore, a key strength of the current study is that it adds to the extant literature on sleep timing across the lifespan, with its findings being specific to Australian adults. The results of the present study could inform future sleep health campaigns specific to the Australian population by providing targeted strategies for different age groups throughout the lifespan to help improve sleep timing. It is important for adults to recognise the influence of lifestyle factors on their sleep timing across their lifespan. For example, Australian adults perform a number of activities in the hour before bed (i.e., watching television, using the internet, and working) that reduce sleep duration and impair daytime functioning [3]. For example, campaigns could educate young Australians about the importance of consistent bed and wake times across the whole week. The addition of questions asking about the minimum amount of sleep required to feel healthy and be in a good mood are novel and another strength of the current study, providing further insight into Australian adults’ perception of the impact sleep has on their overall health. The current findings support a need for a public health campaign focussed on educating the Australian population about the critical role sleep has on our overall health and well-being across the lifespan.

In conclusion, our current findings demonstrate significant variations in sleep timing across the lifespan of Australian adults. Younger adults go to bed and wake up later, especially on weekends; middle-aged adults wake up the earliest; and by the age of 70, the disparity between weekday and weekend wake times decreases. Across all ages, participants reported that on average, the amount of sleep they needed to feel healthy and be in a good mood was less than what they obtained and was below 7 h per night. These findings in Australian adults support international research and emphasise the need for age-specific public health initiatives to support optimal sleep behaviour across the lifespan.

## 4. Materials and Methods

A cross-sectional design was employed to examine the sleep timing of Australian adults across the lifespan. Participants were contacted via telephone to answer a series of questions related to sleep timing. This study formed part of a larger project that was designed to examine individuals’ behaviour in the hours before bedtime.

### 4.1. Participants

A total of 1225 individuals participated in this study. The target population for the research was individuals ≥ 18 years old and living in Australia. The response rate was 35%. Participants were grouped according to their age using 10-year increments. Given that we were examining sleep across the lifespan, we further categorised participants as younger adults, middle-aged adults, and older adults when discussing the key findings. For this study, younger adults were defined as 18–35 years, middle-aged adults were 36–60 years, and older adults were 60 years and above. There was a diverse distribution across the lifespan, with the largest proportion of participants being from the 60–69 age group (22.8%). Please see Table 1 for the participants’ demographic information.

### 4.2. Materials

Participants responded to questions related to demographic information, sleep/wake history, and their perceptions of the sleep required to feel healthy and be in a good mood. The survey also included questions on pre-sleep behaviours, which are not reported in this manuscript (see [15]). Trained interviewers piloted the survey with a randomly selected sample (*n* = 83) to identify and resolve any issues with question order and wording and response coding prior to full data collection.

### 4.3. Sleep/Wake History

Participants were asked to report their sleep/wake behaviours ‘over the past month’, based on the question wording used in the validated measure Pittsburgh Sleep Quality Index [16]. The following questions were used to assess sleep/wake history:

- During the past month, at approximately what time did you wake up on weekdays (Monday–Friday) and weekends (Saturday and Sunday)?
- During the past month, at approximately what time did you go to sleep on weeknights (Sunday–Thursday) and weekends (Friday & Saturday)?
- During the past month, how many hours of sleep did you usually obtain at night?

### 4.4. Health and Mood

Two questions were used to assess individuals’ perceptions about sleep need:

- What is the minimum amount of sleep (hours) that you need each day to feel healthy?
- What is the minimum amount of sleep (hours) that you need each day to be in a good mood?

### 4.5. Procedure

Data collection was carried out through telephone interviews at CQUniversity’s Population Research Laboratory. Trained staff conducted the interviews using a twenty-station Computer-Assisted Telephone Interviewing system on a local network. The interviews took place from 10:30 am to 2:30 pm on Mondays, Wednesdays, and Fridays; from 4:30 pm to 8:30 pm on weekdays; and from 12:00 pm to 4:00 pm on weekends. If the first contact attempt failed, at least five callbacks were made. About half of the participants were reached via mobile phones, with the rest contacted through landlines. Interviewers introduced themselves and asked screening questions to ensure that participants were 18 or older. Participants were informed about the survey duration, voluntary participation, anonymity, the option to skip questions, and the right to withdraw at any time. Interviewers read each question and the response options to the participants, with the average interview lasting 25 min. Data collection was conducted in December 2016.

### 4.6. Data Analysis

The primary aim was to examine if sleep timing (bedtime, wake time, and sleep duration) differed across age groups throughout the lifespan. Analyses were conducted in the R statistical programming environment (Version 4.4.2) [17], with particular use of the *mgcv* package [18] to implement generalised additive models (GAMs). GAMs are similar to standard regression modelling, but the relationship between the predictor and outcome variable is not constrained to being linear, but rather any smooth function. The degree of smoothness is determined by the data, and is parameterised by the effective degrees of freedom, depending on the complexity of the curve. We applied a threshold *p*-value of 0.05 for assessing statistical significance. Each GAM had one predictor, *age*, with the outcome varying: sleep time, wake time, or total duration of the sleep period in hours for both weeknights (Sunday–Thursday) or weekends (Fri/Sat). The minimum sleep period required for maintaining good health and mood was also modelled as a function of age. Taking into consideration that this was part of a large population study and there were seven separate models, each model required approximately 100 observations, resulting in a minimum sample size of 700. Sleep duration was calculated from the below formula, with time (t) in a 24 h format. This approach resolved any potential difficulties in handling 24 h times by the simple assumption that wake time must occur after sleep time.

$$h=\left\{\begin{array}{cc} 24-t_{sleep\;}+t_{wake\;} & \;\mathrm{if}\;t_{sleep\;}>t_{wake\;}\\ t_{wake\;}-t_{sleep\;} & \;\mathrm{if}\;t_{sleep\;}\leq t_{wake\;} \end{array}\right\}$$

## Figures and Tables

**Table 1:** Participant demographics.

		*n*	%
**Gender**	Male	584	47.7
	Female	641	52.3
**Marital Status**	Married/De facto	801	65.3
	Separated/Divorced	115	9.4
	Widowed	70	5.7
	Single	232	18.9
**Age**	18–29 y	153	12.4
	30–39 y	156	12.7
	40–49 y	181	14.8
	50–59 y	251	20.4
	60–69 y	280	22.8
	70–79 y	138	11.3
	80+	66	5.3
**Australian State or**	Australian Capital Territory	36	2.7
**Territory**	New South Wales	392	29.1
	Northern Territory	15	1.1
	Queensland	301	22.3
	South Australia	92	6.8
	Tasmania	33	2.4
	Victoria	345	25.6
	Western Australia	135	10.0

## Data Availability

The raw data supporting the conclusions of this article will be made available by the authors on request.
